# AI-Based Phenotyping of Atrial Fibrillation Through Generative Topographic Mapping: Prospective Murcia Atrial Fibrillation Project III Cohort Study

**DOI:** 10.2196/90502

**Published:** 2026-08-06

**Authors:** Eva Soler Espejo, Yang Chen, María Pilar Ramos-Bratos, Sandra Ortega-Martorell, Iván Olier, José Miguel Rivera-Caravaca, Francisco Marín, Vanessa Roldán, Gregory Y.H. Lip

**Affiliations:** 1 Department of Hematology Hospital Clínico Universitario Virgen de la Arrixaca, University of Murcia, Instituto Murciano de Investigación Biosanitaria Pascual Parrilla (IMIB) El Palmar, Murcia Spain; 2 Liverpool Centre of Cardiovascular Science at University of Liverpool, Liverpool John Moores University and Liverpool Heart and Chest Hospital Liverpool, England United Kingdom; 3 Department of Cardiology Hospital Clínico Universitario Virgen de la Arrixaca, University of Murcia, Instituto Murciano de Investigación Biosanitaria Pascual Parrilla (IMIB), CIBERCV El Palmar, Murcia Spain; 4 Artificial Intelligence and Digital Technologies Research Institute, Liverpool John Moores University Liverpool, England United Kingdom; 5 Faculty of Nursing University of Murcia, Instituto Murciano de Investigación Biosanitaria Pascual Parrilla (IMIB), CIBERCV Murcia Spain; 6 Department of Clinical Medicine Aalborg University Aalborg, North Denmark Denmark; 7 University of Białystok Białystok, Podlasie Poland

**Keywords:** AF, AI, artificial intelligence, atrial fibrillation, clinical outcomes, generative topographic mapping, phenotypes

## Abstract

**Background:**

The clinical heterogeneity of atrial fibrillation (AF) challenges current classifications and risk scores, limiting their real-world applicability. AI-driven methods may enhance phenotyping and risk stratification.

**Objective:**

This study aimed to apply a generative topographic mapping (GTM)–based clustering approach to a large, real-world, prospective AF cohort to identify clinically relevant phenotypes and assess their associations with clinical outcomes.

**Methods:**

We conducted a prospective observational cohort study including consecutive adult outpatients with newly diagnosed AF who initiated oral anticoagulation between January 2016 and November 2021. Cardiometabolic risk was modeled as a multidimensional construct integrating cardiovascular and metabolic comorbidities. GTM was applied to project high-dimensional clinical data into a low-dimensional latent space, enabling probabilistic patient representation and visualization of phenotypic structure. Unsupervised hierarchical clustering using Ward’s method was performed on the latent space to identify AF phenotypes, and patients were assigned to clusters based on their highest posterior probability. Clinical outcomes, including thromboembolic events, major bleeding, major adverse cardiovascular events (MACE), cardiovascular death, and all-cause death, were assessed over a maximum follow-up of 2 years. Nonfatal outcomes were analyzed using Fine-Gray competing-risk models and reported as subdistribution hazard ratios (sHRs), whereas cardiovascular and all-cause death were analyzed using adjusted Cox proportional hazards models and reported as adjusted hazard ratios (aHRs).

**Results:**

Among 3259 patients with AF (median age 77, IQR 70-83 years; 1721/3259, 52.8% female; mean follow-up 1.81 years), four phenotypes emerged: (1) older with highest cardiometabolic burden, (2) older with lower cardiometabolic risk, (3) comparatively younger with intermediate-high cardiometabolic risk, and (4) comparatively younger with intermediate cardiometabolic risk. Compared with phenotype 1, phenotype 4 demonstrated lower risks of thromboembolic events (sHR 0.70, 95% CI 0.50-0.98), major bleeding (sHR 0.61, 95% CI 0.42-0.89), and MACE (sHR 0.67, 95% CI 0.47-0.94). Regarding cardiovascular and all-cause death, all phenotypes demonstrated a lower risk compared with phenotype 1: phenotype 2 (aHR 0.52, 95% CI 0.33-0.85; and aHR 0.66, 95% CI 0.50-0.87, respectively), phenotype 3 (aHR 0.57, 95% CI 0.32-0.99; and aHR 0.44, 95% CI 0.30-0.65, respectively), and phenotype 4 (aHR 0.49, 95% CI 0.33-0.72; and aHR 0.56, 95% CI 0.44-0.71, respectively). However, these associations were attenuated after further adjustment for age, sex, and major comorbidities, with phenotype 4 retaining a significant association with lower cardiovascular death and phenotypes 3 and 4 retaining significant associations with lower all-cause death.

**Conclusions:**

GTM-based clustering analysis identified prognostically distinct AF phenotypes, highlighting the potential of machine learning approaches to support personalized AF care. Further external validation is needed to establish the generalizability of these findings.

## Introduction

Atrial fibrillation (AF) is the most common sustained cardiac arrhythmia, affecting 1% to 2% of the global population, and is expected to increase due to aging populations and the rising burden of cardiovascular risk factors [1]. AF poses a significant public health challenge due to its strong associations with thromboembolic events, anticoagulation-related bleeding, cardiovascular complications, and increased mortality [2,3]. Despite its high prevalence, AF remains clinically heterogeneous, with patients presenting diverse profiles, comorbidities, and risk factors [4].

Traditional risk scores and classifications based on arrhythmia patterns or subtypes often fail to capture this complexity, limiting their effectiveness for accurate risk stratification and personalized treatment [5]. Given this heterogeneity, classifying patients into clinically relevant phenotypes could enhance risk assessment and support more individualized therapeutic strategies [6].

Previous methods for identifying AF phenotypes, such as hierarchical clustering [7,8] and k-prototype [5] algorithms, have limitations in fully capturing patient heterogeneity. These approaches are often constrained by predefined assumptions and may overlook subtle relationships between clinical variables [9]. In contrast, a novel machine learning methodology [10] that uses generative topographic mapping (GTM) offers a more flexible, data-driven approach. This methodology [10] facilitates the identification and visualization of complex patient distributions in a probabilistic and interpretable manner [11], enabling the discovery of clinically meaningful phenotypes [10,12]. To date, GTM has not been applied to identify phenotypes of AF in the context of a prospective registry.

In this study, we applied a GTM-based clustering approach to a large, real-world, prospective AF cohort to identify clinically relevant phenotypes and assess their associations with clinical outcomes.

## Methods

### Overview

This prospective observational study, the Murcia Atrial Fibrillation Project III (MAFP-III), was based on a consecutive registry of outpatients with a recent diagnosis of AF who were treatment-naïve to oral anticoagulation (OAC) and initiated therapy with either vitamin K antagonists (VKAs) or direct-acting OACs (DOACs). Patients were enrolled from 2 anticoagulation clinics at 2 tertiary hospitals (Murcia, Spain) between January 1, 2016, and November 30, 2021. Only adult patients (aged ≥18 years) were eligible for inclusion. Exclusion criteria included patients with prosthetic heart valves, rheumatic mitral valves, or other severe valvular diseases. Baseline data were collected prospectively according to a predefined protocol, and patients were followed for clinical outcomes. The GTM-based phenotypic clustering and the evaluation of primary outcomes were conducted retrospectively as exploratory analyses once follow-up was complete; these analyses were not prespecified in the original study protocol.

### Data Extraction and Definition of Cardiometabolic Risk

Data extracted at baseline comprised sociodemographic and anthropometric data, comorbidities, laboratory parameters, echocardiographic features, and concomitant therapies. Additionally, stroke risk assessed by CHA₂DS₂-VASc (Congestive heart failure, Hypertension, Age ≥ 75 [doubled score], Diabetes, Stroke [doubled], Vascular disease, Sex category [female]) [13] and CHA₂DS₂-VA (Congestive heart failure, Hypertension, Age ≥ 75 [doubled score], Diabetes, Stroke [doubled], Vascular disease) [14], and bleeding risk assessed by HAS-BLED (Hypertension, Abnormal renal and liver function, Stroke, Bleeding, Labile INR, Elderly, Drugs or alcohol) [15] were evaluated at baseline.

In this study, cardiometabolic risk was conceptualized as a multidimensional construct reflecting the cumulative burden of cardiovascular and metabolic dysfunction relevant to AF prognosis. The GTM model was trained using structured demographic, laboratory, hemodynamic, and echocardiographic variables. Clinical comorbidities and lifestyle-related factors, including hypertension; diabetes mellitus; heart failure; history of stroke, transient ischemic attack (TIA), and thromboembolism; vascular disease (coronary artery disease and/or peripheral artery disease); renal impairment; hypercholesterolemia; chronic obstructive pulmonary disease (COPD) or obstructive sleep apnea (OSA); liver disease; smoking habit; and alcohol use disorder, were evaluated as additional investigative variables and projected post hoc onto the latent space to support interpretation of the derived phenotypic clusters [16,17].

### AI-Based Methodology to Generate Reliable Phenotypes

#### GTM

To explore phenotypic heterogeneity within the MAFP-III cohort of patients with AF, we applied GTM, a nonlinear dimensionality reduction technique well-suited for complex, high-dimensional biomedical data [11]. Following previous approaches [10], the model was trained on a reference data matrix comprising patient-level variables, including demographics, blood count parameters, diabetes risk markers, renal and liver function indicators, cholesterol markers, arterial blood pressure, and echocardiographic measurements. These training variables corresponded exclusively to the predefined modeling variables presented in the cohort baseline characteristics. The manifold structure was optimized in latent space using the expectation-maximization algorithm, resulting in a low-dimensional projection of the data.

Clinical comorbidities and other exploratory variables were not directly included during GTM training but were instead projected post hoc onto the latent space to facilitate phenotypic interpretation.

Each patient’s position in the latent space was characterized by a responsibility matrix, representing posterior probabilities of association with each of the nodes in the GTM grid. This enabled a soft assignment of patients across the latent space and supported a probabilistic interpretation of patient similarity.

To enhance cluster interpretability, additional variables, such as demographic characteristics and comorbidities not used in model training, were projected post hoc onto the latent space. This supplementary mapping provided further insight into potential phenotypic factors beyond the modeling variables.

To identify the optimal hyperparameter configuration for the GTM model, we performed an exhaustive grid search over a range of values for 3 key parameters: number of latent clusters, the penalization term used to regulate the mapping process, and the number of radial basis functions. For each parameter set, the GTM model was trained using 10-fold cross-validation. Model performance on each fold was evaluated using the negative log-likelihood (NLL) of the test data under the fitted model. Among successful runs, the average and SD of the NLLs across folds were recorded. The top-performing configurations, based on the lowest mean NLL and lowest SD, were retained for further analysis. Finally, the optimal parameter combination included a 15 × 15 latent space grid, 196 radial basis functions arranged in a 14 × 14 layout, and a regularization term of 1.

#### Hierarchical Clustering and Phenotypic Stratification

Following GTM projection, we performed unsupervised hierarchical clustering using Ward’s linkage method [18] and Euclidean distance to identify distinct phenotypic subgroups. Dendrograms were used to determine the optimal number of macroclusters [10]. Each patient was assigned to a cluster by mapping their most probable latent node (modal responsibility) to the corresponding cluster label, which was then reintegrated into the dataset for downstream analyses.

#### Cluster Propagation to Individual Patients

Phenotypic cluster labels were propagated to individual patients based on the cluster identity of their modal latent node—the node to which they were most strongly associated. This approach maintained the probabilistic nature of the GTM while enabling direct assignment of cluster labels for clinical and statistical interpretation. The assigned labels were stored as a new variable in the dataset.

#### Software and Implementation

All analyses were conducted using Python (version 3.12.7) in the Jupyter Notebook environment. Data preprocessing and transformation were performed using standard scientific libraries, including “*pandas*,” “*numpy*,” and “*scikit-learn*.” The code from the original methodology to derive meaningful clinical phenotypes used in this study was freely available on Zenodo [10]. The GTM model was implemented with the “*ugtm*” package. Visualizations, such as latent space projections, hierarchical dendrograms, cluster assignments, and responsibility maps, were generated using packages including “*matplotlib*,” “*seaborn*,” and “*plotly*.” Results and cluster assignments were exported for further integration with external platforms for statistical and clinical analysis.

### Primary Clinical Outcomes and Follow-Up

The primary end points included (1) any thromboembolic event, defined as a composite of ischemic stroke, TIA, or venous thromboembolism; (2) major bleeding based on the 2005 International Society on Thrombosis and Haemostasis (ISTH) criteria [19]; (3) major adverse cardiovascular events (MACE) comprising myocardial infarction, ischemic stroke or TIA, or cardiovascular death; (4) cardiovascular death; and (5) all-cause death. These outcomes were chosen because cardiometabolic comorbidities are key determinants of thromboembolic, bleeding, and overall cardiovascular risk in anticoagulated AF and have a well-established impact on both cardiovascular and all-cause prognosis [2]. The maximum follow-up period was set to 2 years for all participants.

### Statistical Analysis

Quantitative variables were presented as mean (SD) or median (IQR), when appropriate. Differences in quantitative variables between the clusters were assessed using ANOVA or the Kruskal-Wallis test for nonparametric data. Post hoc pairwise comparisons were performed using the Dunn test with Bonferroni correction, using phenotype 1 as the reference group. Categorical variables were expressed as absolute frequencies and percentages, and differences between proportions were compared using the Pearson chi-square test. Additional pairwise post hoc comparisons for categorical variables were also performed using phenotype 1 as the reference group.

Adjusted Cox proportional hazards regression models were used to assess the independent association between phenotype membership and time-to-event outcomes. Multivariable models incorporated concomitant treatments, including OAC type (DOACs and VKAs), antiarrhythmics, angiotensin-converting enzyme inhibitors, angiotensin II receptor blockers, calcium channel blockers, antilipemic agents, beta-blockers, diuretics, oral hypoglycemic agents, insulins, and antiplatelet therapy. Given the relatively high all-cause death observed during follow-up, additional competing-risk analyses were performed for nonfatal outcomes (thromboembolic events, major bleeding, and MACE) using Fine-Gray subdistribution hazard models, with death treated as a competing event. Cardiovascular death and all-cause death were analyzed using conventional Cox proportional hazards models.

As a sensitivity analysis, additional multivariable models were constructed adjusting for age; sex; hypertension; diabetes mellitus; heart failure; and history of stroke, TIA, thromboembolism, and vascular disease.

All covariates were included simultaneously using the forced-entry (Enter) method, based on prespecified clinical relevance rather than statistical selection thresholds; no stepwise selection procedure was applied.

Results from Cox regression analyses were reported as adjusted hazard ratios (aHRs), whereas competing-risk analyses were reported as subdistribution hazard ratios (sHRs), both with 95% CIs.

Event-free survival was analyzed using Kaplan-Meier curves, with differences assessed using the log-rank test.

A *P* value of <.05 was considered statistically significant. Statistical analyses were carried out using R software (version 4.4.0; R Foundation for Statistical Computing), SPSS (version 25.0; IBM Corp), Stata statistical software (version 16.0; StataCorp), and MedCalc (version 16.4.3; MedCalc Software bvba) for Microsoft Windows.

### Ethical Considerations

The study protocol was approved by the Ethics Committee of the University Hospital Morales Meseguer (reference number EST:20/16) and the University Hospital Virgen de la Arrixaca (reference number 2020-11-12-HCUVA). The study was conducted in accordance with the ethical principles outlined in the Declaration of Helsinki and its subsequent amendments.

All participants provided written informed consent prior to inclusion in the study. The original informed consent covered the collection and analysis of clinical data for research purposes.

All data were handled in accordance with applicable data protection regulations. Patient data were anonymized and deidentified prior to analysis to ensure privacy and confidentiality. No financial compensation was provided to participants for their involvement in this study. No identifiable individual participant data or images are included in this manuscript.

## Results

### Overview

We included 3259 patients with AF (1721/3259, 52.8% female; median age 77, IQR 70-83 years), with a median CHA_2_DS_2_-VASc score of 4 (IQR 3-5), a median CHA_2_DS_2_-VA score of 3 (IQR 3-5), and a median HAS-BLED score of 3 (IQR 2-4). Within this cohort, a total of 1050 (32.2%) patients were treated with VKAs, and 2209 (67.8%) with DOACs. A summary of the variables used for modeling and the post hoc investigative variables is provided in Table 1. Pairwise post hoc comparison *P* values, with phenotype 1 as the reference group, are presented in Table S1 in Multimedia Appendix 1.

**Table 1:** Baseline clinical characteristics of the four generative topographic mapping–derived phenotypes identified in the Murcia Atrial Fibrillation Project III, a prospective observational cohort of anticoagulated patients with newly diagnosed AF recruited in Murcia, Spain, between January 2016 and November 2021.

Variables				Phenotype 1 (n=619)		Phenotype 2 (n=677)		Phenotype 3 (n=353)	Phenotype 4 (n=1610)			*P* value
**Modeling variables**												
	**Demographics, median (IQR)**											
		Weight (kg)	82 (73-92)		74 (65-83)		108 (95-119)			80 (70-90)	<.001	
		Abdominal circumference (cm)	109 (102-117)		100 (92-107)		124 (116-133)			106 (97-112)	<.001	
		Body fat (%)	45 (37-51)		39 (34-46)		54 (42-57)			41 (35-47)	<.001	
	**Blood count parameters, median (IQR)**											
		Hemoglobin (g/dL)	13 (11-14)		13 (12-14)		14 (12-15)			14 (13-15)	<.001	
		Leukocyte count (×10^9^/L)	8 (7-10)		7 (6-8)		8 (7-9)			8 (7-9)	<.001	
		Platelet count (×10^9^/L)	225 (190-275)		213 (174-253)		219 (186-255)			214 (178-256)	<.001	
	**Diabetes risk markers, median (IQR)**											
		Glucose (mg/dL)	145 (113-192)		100 (90-114)		108 (93-126)			105 (94-126)	<.001	
		HbA_1c_^a^ (%)	7.5 (6.4-8.2)		5.9 (5.6-6.4)		6.0 (5.7-6.6)			6.1 (5.8-6.5)	<.001	
	**Renal function, median (IQR)**											
		Creatinine (mg/dL)	1.1 (0.9-1.7)		0.9 (0.7-1.2)		0.9 (0.8-1.2)			1 (0.8-1.1)	<.001	
	**Liver function, median (IQR)**											
		Aspartate aminotransferase (U/L)	18 (14-21)		18 (14-22)		20 (18-27)			21 (17-26)	<.001	
		Alanine aminotransferase (U/L)	15 (12-20)		13 (10-18)		19 (15-27.5)			19 (14-25)	<.001	
	**Cholesterol markers, median (IQR)**											
		Cholesterol (mg/dL)	134 (113-164)		147 (120-177)		159 (144-179)			169 (143-200)	<.001	
		HDL^b^ cholesterol (mg/dL)	41 (32-46)		54 (44-63)		47 (41-56)			48 (40-58)	<.001	
		Triglycerides (mg/dL)	124 (96-165)		82 (66-110)		112 (90-150)			125 (99-185)	<.001	
	**Arterial blood pressure, median (IQR)**											
		Systolic BP^c^ (mm Hg)	133 (120-145)		130 (120-140)		137 (127-148)			137 (125-150)	<.001	
		Diastolic BP (mm Hg)	70 (60-77)		71 (66-80)		79 (70-85)			77 (70-85)	<.001	
	**Echocardiographic markers, median (IQR)**											
		Left ventricular ejection fraction (%)	57 (50-65)		59 (55-65)		58 (47-65)			59 (49-63)	.12	
**Additional investigative variables**												
	**Demographics**											
		Age (years), median (IQR)	79 (74-84)		79 (73-84)		74 (67-81)			75 (68-82)	<.001	
		Sex (female), n (%)	345 (55.7)		375 (55.4)		225 (63.7)			776 (48.2)	<.001	
		BMI (kg/m^2^), median (IQR)	30 (27-33)		27 (24-30)		38 (35-42)			29 (27-32)	<.001	
	**Type of atrial fibrillation, n (%)**											
		Persistent	401 (64.8)		434 (64.1)		248 (70.3)			979 (60.8)	.006	
		Paroxysmal	218 (35.2)		243 (35.9)		105 (29.7)			631 (39.2)	.006	
	**Comorbidities, n (%)**											
		Hypertension	552 (89.2)		532 (78.6)		328 (92.9)			1354 (84.1)	<.001	
		Diabetes mellitus	426 (68.8)		167 (24.6)		146 (41.4)			530 (32.9)	<.001	
		Heart failure	167 (27.0)		100 (14.8)		95 (26.9)			325 (20.2)	<.001	
		History of stroke, TIA^d^, or thromboembolism	158 (25.5)		177 (26.1)		77 (21.8)			334 (20.7)	.01	
		Vascular disease^e^	176 (28.4)		139 (20.5)		49 (13.9)			310 (19.3)	<.001	
		Renal impairment	241 (38.9)		151 (22.3)		60 (17.0)			260 (16.1)	<.001	
		Hypercholesterolemia	400 (64.6)		330 (48.7)		198 (56.1)			926 (57.5)	<.001	
		COPD^f^ or OSA^g^	160 (25.8)		119 (17.6)		132 (37.4)			313 (19.4)	<.001	
		History of relevant bleeding	376 (60.7)		306 (45.2)		133 (37.7)			454 (28.2)	<.001	
		Liver disease	29 (4.7)		28 (4.1)		25 (7.1)			70 (4.3)	.14	
		History of cancer	92 (14.9)		114 (16.8)		41 (11.6)			209 (13.0)	.047	
		Smoking habit	134 (21.6)		175 (25.8)		70 (19.8)			370 (23.0)	.13	
		Alcoholism	41 (6.6)		61 (9.0)		27 (7.6)			153 (9.5)	.15	
	**Concomitant treatment, n (%)**											
		Direct-acting oral anticoagulation	417 (67.4)		566 (83.6)		201 (56.9)			1025 (63.7)	<.001	
		Vitamin K antagonist	202 (32.6)		111 (16.4)		152 (43.1)			585 (36.3)	<.001	
		Antiarrhythmics	89 (14.4)		53 (15.0)		108 (16.0)			242 (15.0)	.89	
		Angiotensin-converting enzyme inhibitors	166 (26.8)		172 (25.4)		92 (26.1)			374 (23.2)	.23	
		Angiotensin II receptor blockers	269 (43.5)		271 (40.0)		188 (53.3)			704 (43.8)	<.001	
		Calcium channel blockers	215 (34.7)		182 (26.9)		104 (29.5)			449 (27.9)	.007	
		Beta-blockers	397 (64.1)		422 (62.3)		239 (67.7)			1078 (67.0)	.12	
		Diuretics	431 (69.6)		375 (55.4)		230 (65.2)			850 (52.8)	<.001	
		Antilipemic agents	393 (63.5)		337 (49.8)		194 (55.0)			871 (54.1)	<.001	
		Oral hypoglycemic agents	323 (52.2)		139 (20.5)		105 (29.7)			405 (25.2)	<.001	
		Insulins	142 (22.9)		35 (5.2)		21 (5.9)			96 (6.0)	<.001	
		Antiplatelet therapy	119 (19.2)		81 (12.0)		41 (11.6)			214 (13.3)	<.001	
**Stroke and bleeding scores, median (IQR)**												
	CHA_2_DS_2_-VASc		5 (4-6)		4 (3-5)		4 (3-5)			4 (3-5)	<.001	
	CHA_2_DS_2_-VA		4 (3-5)		3 (3-5)		3 (2-5)			3 (2-4)	<.001	
	HAS-BLED		4 (3-5)		3 (2-4)		3 (2-4)			3 (2-4)	<.001	

^a^HbA_1c_: glycated hemoglobin.

^b^HDL: high-density lipoprotein.

^c^BP: blood pressure.

^d^TIA: transient ischemic attack.

^e^Includes coronary artery disease and/or peripheral artery disease.

^f^COPD: chronic obstructive pulmonary disease.

^g^OSA: obstructive sleep apnea.

### Topographic Representation of Modeling Variables

**Figure 1:**
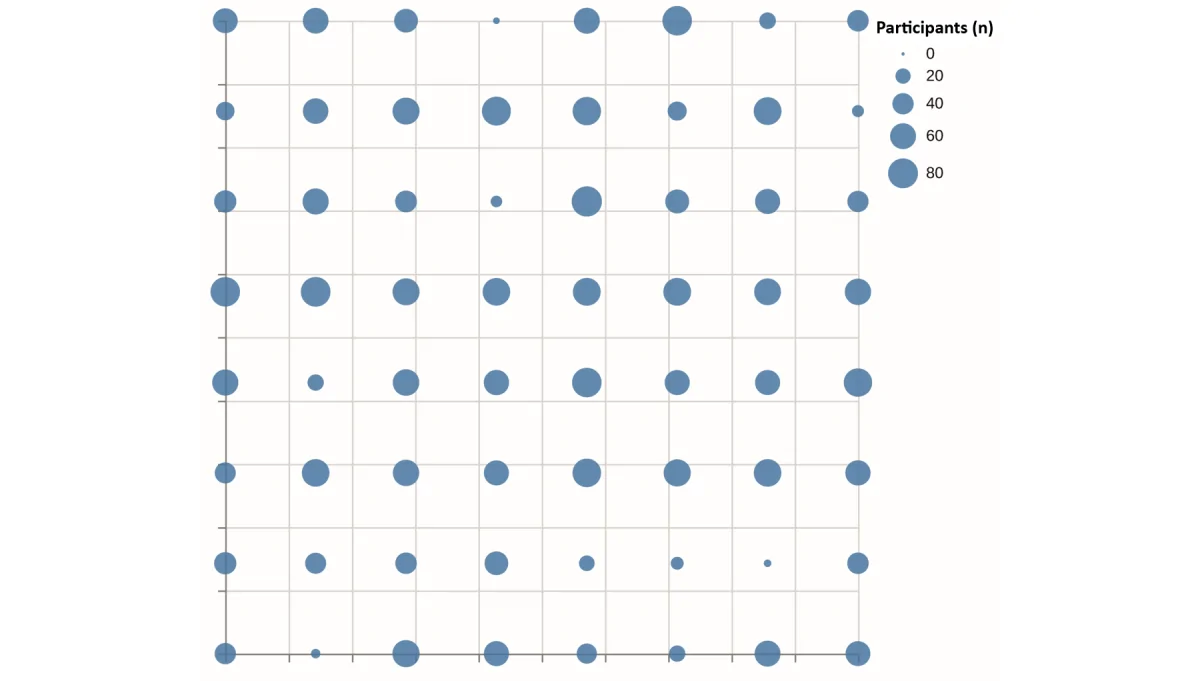
Generative topographic mapping (GTM) latent-space membership map showing the probabilistic distribution of patients with atrial fibrillation from the Murcia Atrial Fibrillation Project III (MAFP-III) cohort across the 2D latent manifold generated using the GTM-based phenotyping framework. The size of each cluster reflects the number of patients allocated to it.

**Figure 2:**
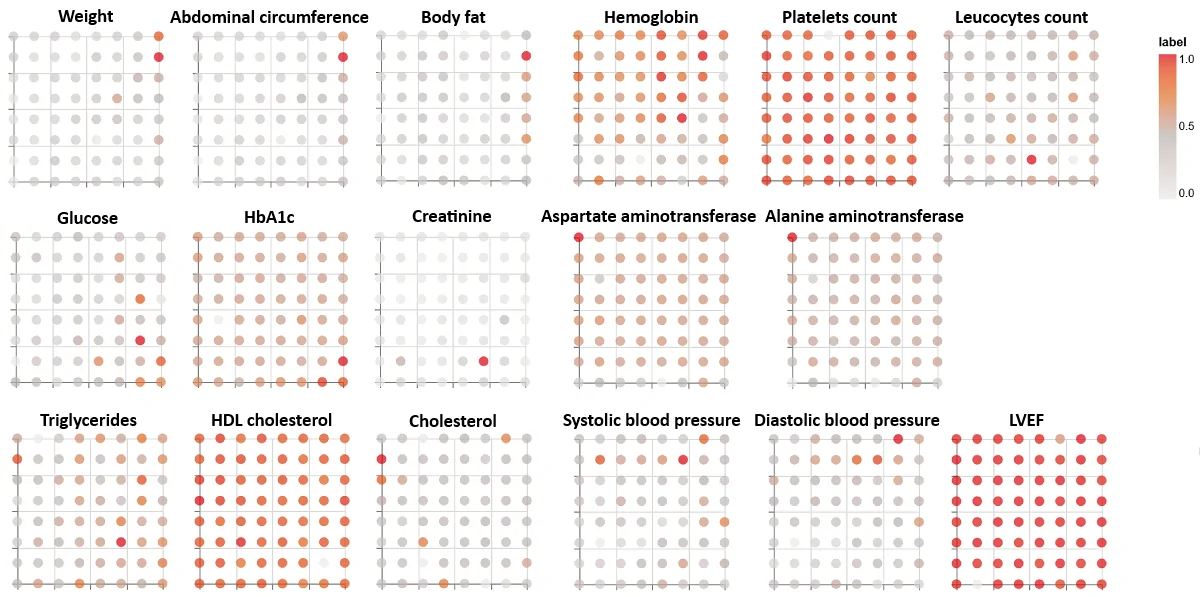
Generative topographic mapping (GTM) reference vector maps illustrating the distribution of modeling variables across the latent space in the Murcia Atrial Fibrillation Project III (MAFP-III) cohort. Variables included in GTM model training are grouped according to clinically relevant domains, including demographics, blood count parameters, diabetes risk markers, renal and liver function, cholesterol markers, arterial blood pressure, and echocardiographic features. Color intensity reflects the relative magnitude of each variable across latent-space regions. HbA1c: glycated hemoglobin; HDL: high-density lipoproteins; LVEF: left ventricular ejection fraction.

The reference vectors derived from the GTM model trained on this cohort are shown in Figure 1. These maps illustrate the distribution of core modeling variables across the latent space, grouped into clinically relevant domains (demographics, blood count parameters, diabetes risk markers, renal and liver function, cholesterol markers, arterial blood pressure, and echocardiographic features). Each point corresponds to a node in the GTM grid, maintaining the probabilistic structure of the model (Figure 2). A red gradient indicates the intensity of each variable: darker red denotes higher values, while lighter areas reflect lower levels. All variables displayed were included in model training and contributed directly to the identification of phenotypic subgroups.

### Mapping of Additional Investigative Variables

**Figure 3:**
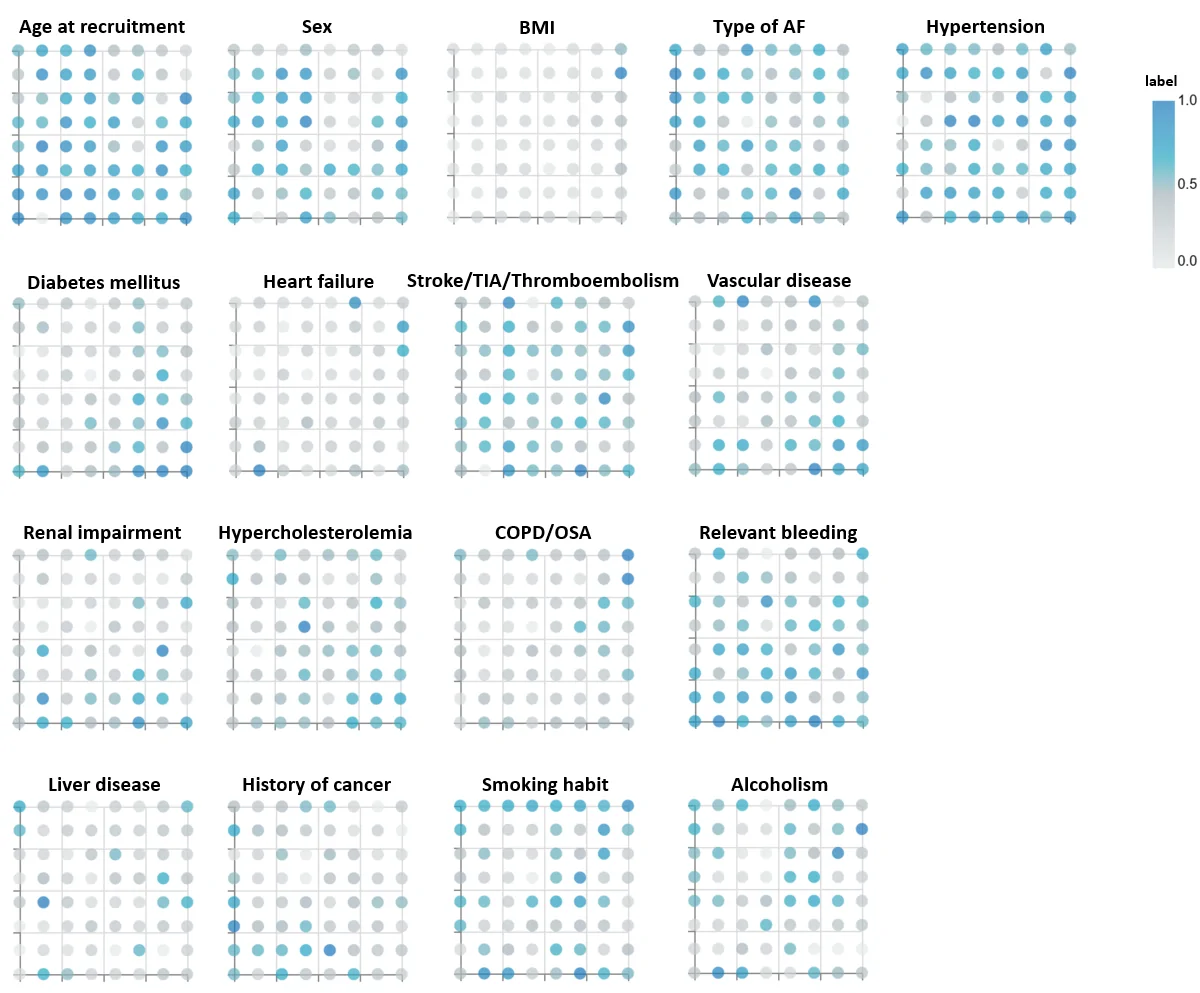
Post hoc projection maps showing the distribution of additional investigative clinical variables across the generative topographic mapping (GTM) latent space in the Murcia Atrial Fibrillation Project III (MAFP-III) cohort. These variables were not included during GTM model training but were projected onto the latent manifold to facilitate interpretation of the derived phenotypic clusters. Color intensity reflects the relative distribution of each variable across latent-space regions. AF: atrial fibrillation; COPD/OSA: chronic obstructive pulmonary disease/obstructive sleep apnea; TIA: transient ischemic attack.

Figure 3 shows the distribution of additional clinical variables not included in model training but mapped post hoc to help interpret the phenotypic clusters. These are visualized using a gray–teal color scale, where deeper teal shades correspond to higher average values within each microcluster. Each node reflects the mean value of the variable among patients most strongly associated with that region. This exploratory mapping helps identify clinically meaningful patterns not directly involved in model construction but potentially relevant to the characterization of phenotypic subgroups.

### Hierarchical Clustering and Identification of AF phenotypes

#### Overview

**Figure 4:**
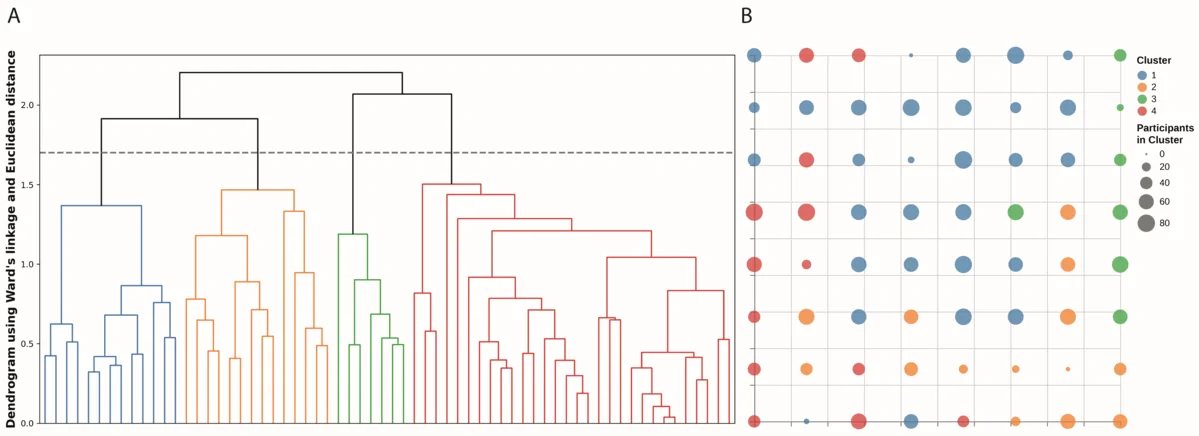
Hierarchical clustering and phenotypic stratification of patients from the Murcia Atrial Fibrillation Project III (MAFP-III) cohort using the generative topographic mapping (GTM) framework. (A) Dendrogram generated using Ward’s linkage method applied to GTM reference vectors to identify phenotypic macroclusters. (B) Visual representation of the 4 derived phenotypic regions projected onto the GTM latent space.

Hierarchical clustering of the GTM reference vectors using Ward’s minimum variance method identified 4 distinct macroclusters, as shown in Figure 4A. The optimal number of clusters was determined by applying a threshold cutoff of 1.70, corresponding to the approximate median Y-axis linkage distance, which maximized separation while preserving clinical interpretability. These clusters were subsequently mapped onto the GTM latent space, defining 4 phenotypic regions across the manifold, as depicted in Figure 4B, each representing a unique clinical pattern. Baseline characteristics for each phenotype are summarized in Table 1.

#### Phenotype 1: Older Patients With the Highest Cardiometabolic Burden

Phenotype 1 included 619 (19%) patients with a median age of 79 (IQR 74-84) years, predominantly female (345/619, 55.7%). This phenotype was characterized by the highest prevalence of diabetes (426/619, 68.8%), heart failure (167/619, 27%), vascular disease (176/619, 28.4%), renal impairment (241/619, 38.9%), hypercholesterolemia (400/619, 64.6%), and relevant bleeding history (376/619, 60.7%). Additionally, it showed the second highest prevalence of hypertension (552/619, 89.2%); stroke, TIA, and thromboembolism (158/619, 25.5%); COPD and OSA (160/619, 25.8%); and history of cancer (92/619, 14.9%).

#### Phenotype 2: Older Patients With Lower Cardiometabolic Risk

Phenotype 2 included 677 (20.8%) patients with a median age of 79 (IQR 73-84) years, predominantly female (375/677, 55.4%). This group had lower proportions of hypertension (532/677, 78.6%), diabetes (167/677, 24.6%), heart failure (100/677, 14.8%), liver disease (28/677, 4.1%), hypercholesterolemia (330/677, 48.7%), and COPD and OSA (119/677, 17.6%) compared to the other phenotypes.

#### Phenotype 3: Comparatively Younger Patients With Intermediate-High Cardiometabolic Risk

Phenotype 3 was the smallest group, comprising 353 (10.8%) patients with a median age of 74 (IQR 67-81) years, predominantly female (225/353, 63.7%). This phenotype had the highest BMI (median 38, IQR 35-42 kg/m^2^) and the greatest prevalence of hypertension (328/353, 92.9%), COPD and OSA (132/353, 37.4%), and liver disease (25/353, 7.1%). It also had the lowest prevalence of vascular disease (49/353, 13.9%) and smoking habit (70/353, 19.8%). This group had the highest prevalence of persistent AF (248/353, 70.3%).

#### Phenotype 4: Comparatively Younger Patients With Intermediate Cardiometabolic Risk

Phenotype 4 was the largest group, comprising 1610 (49.4%) patients with a median age of 75 (IQR 68-82) years, predominantly male (834/1610, 51.8%). This phenotype exhibited intermediate rates of hypertension (1354/1610, 84.1%), diabetes (530/1610, 32.9%), heart failure (325/1610, 20.2%), vascular disease (311/1610, 19.3%), COPD and OSA (312/1610, 19.4%), and smoking (370/1610, 23%) compared with the other phenotypes. It also had the lowest prevalence of stroke, TIA, and thromboembolism (333/1610, 20.7%); renal impairment (259/1610, 16.1%); and history of relevant bleeding (454/1610, 28.2%), along with the highest prevalence of alcoholism (153/1610, 9.5%).

### Interpreting the Visualizations

The GTM model assigns each patient a probability of belonging to the various nodes within the latent space, allowing for a detailed visualization of their position on the map. Although each individual may have some probability of belonging to multiple nodes, the final cluster assignment is based on the node with the highest probability.

**Figure 5:**
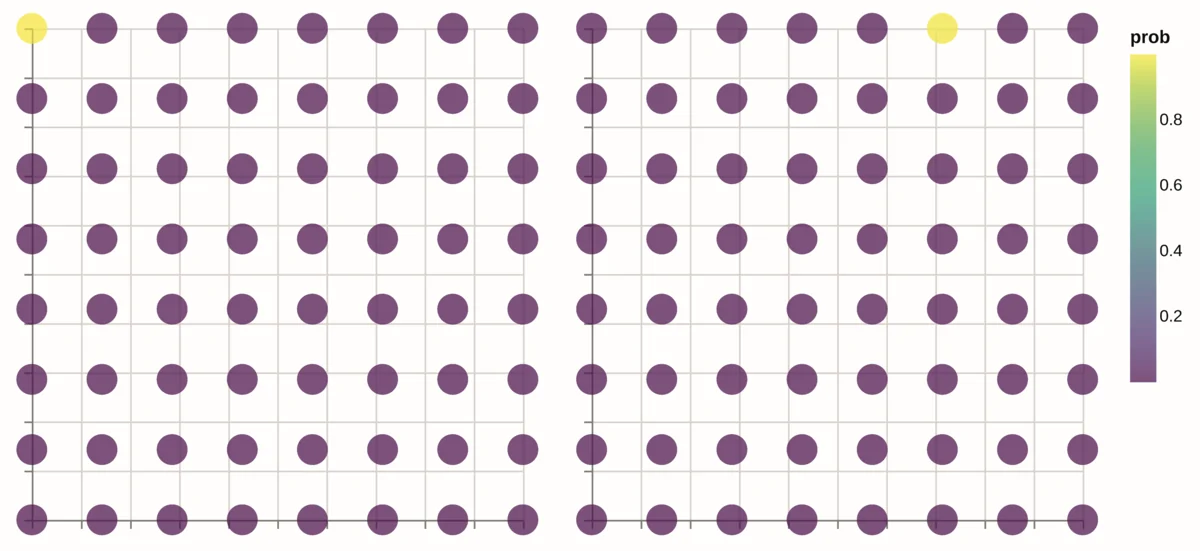
Probabilistic patient assignment within the generative topographic mapping (GTM) latent space in the Murcia Atrial Fibrillation Project III (MAFP-III) cohort. Maps illustrate the probability distributions of 2 representative patients across latent-space nodes, demonstrating the probabilistic nature of GTM-based phenotypic classification and the degree of membership uncertainty across neighboring regions. prob: probability.

In our data, randomly selected patients showed high assignment probabilities (Figure 5), indicating well-defined and robust classifications. This probabilistic distribution not only supports the reliability of the model but also allows the identification of individuals near cluster boundaries, which could have clinical relevance.

### Primary Clinical Outcomes Among Phenotypes

Over a mean follow-up of 1.81 (SD 0.48) years, 246 patients (7.6%) experienced a thromboembolic event, with major bleeding occurring in 196 (6%), MACE in 350 (12.7%), cardiovascular death in 159 (4.9%), and all-cause death in 437 (13.4%). The incidence of all clinical outcomes differed significantly across phenotypes, with a progressive increase in adverse events from phenotype 1 onwards (*P*=.008 for any thromboembolic event, *P*=.006 for major bleeding, and *P*<.001 for the remaining outcomes).

Cox regression analyses, adjusted for concomitant treatments (OAC type [DOACs and VKAs], antiarrhythmics, angiotensin-converting enzyme inhibitors, angiotensin II receptor blockers, calcium channel blockers, antilipemic agents, beta-blockers, diuretics, oral hypoglycemic agents, insulins, and antiplatelet therapy), are summarized in Table 2. All comparisons were performed using phenotype 1 as the reference group.

**Table 2:** Association between generative topographic mapping–derived atrial fibrillation phenotypes and clinical outcomes in the Murcia Atrial Fibrillation Project III cohort. Adjusted Fine-Gray competing-risk models were used for nonfatal outcomes (thromboembolic events, major bleeding, and MACE^a^), with death treated as a competing event, whereas adjusted Cox proportional hazards models were used for cardiovascular death and all-cause death.

Outcome		Adjusted model^b^	
		HR^c^ (95% CI)	*P* value
**Any thromboembolic event**			
	Phenotype 1 (n=619; reference)		
	Phenotype 2 (n=677)	0.78 (0.52-1.17)	.23
	Phenotype 3 (n=353)	0.61 (0.37-1.02)	.06
	Phenotype 4 (n=1610)	0.70 (0.50-0.98)	.04
**Major bleeding**			
	Phenotype 1 (n=619; reference)		
	Phenotype 2 (n=677)	0.95 (0.63-1.43)	.08
	Phenotype 3 (n=353)	0.80 (0.48-1.33)	.38
	Phenotype 4 (n=1610)	0.61 (0.42-0.89)	.01
**MACE**			
	Phenotype 1 (n=619; reference)		
	Phenotype 2 (n=677)	0.68 (0.45-1.03)	.07
	Phenotype 3 (n=353)	0.61 (0.37-1.01)	.05
	Phenotype 4 (n=1610)	0.67 (0.47-0.94)	.02
**Cardiovascular death**			
	Phenotype 1 (n=619; reference)		
	Phenotype 2 (n=677)	0.52 (0.33-0.85)	.008
	Phenotype 3 (n=353)	0.57 (0.32-0.99)	.048
	Phenotype 4 (n=1610)	0.49 (0.33-0.72)	<.001
**All-cause death**			
	Phenotype 1 (n=619; reference)		
	Phenotype 2 (n=677)	0.66 (0.50-0.87)	.003
	Phenotype 3 (n=353)	0.44 (0.30-0.65)	<.001
	Phenotype 4 (n=1610)	0.56 (0.44-0.71)	<.001

^a^MACE: major adverse cardiovascular events.

^b^Adjusted model by concomitant treatment (oral anticoagulation type [direct-acting oral anticoagulants and vitamin K antagonists], antiarrhythmics, angiotensin-converting enzyme inhibitors, angiotensin II receptor blockers, calcium channel blockers, antilipemic agents, beta-blockers, diuretics, oral hypoglycemic agents, insulins and antiplatelet therapy).

^c^HR: hazard ratio; subdistribution HRs are reported for nonfatal outcomes, while adjusted HRs are reported for cardiovascular and all-cause death.

For thromboembolic events, compared to phenotype 1, phenotype 4 was associated with a significantly lower risk (sHR 0.70, 95% CI 0.50-0.98; *P*=.04), whereas phenotypes 2 and 3 showed nonsignificant trends toward risk reduction.

Regarding major bleeding, only phenotype 4 demonstrated a significantly lower risk compared to phenotype 1 (sHR 0.61, 95% CI 0.42-0.89; *P*=.01), while the associations observed for phenotypes 2 and 3 did not reach statistical significance.

For MACE, phenotype 4 was also associated with a significantly reduced risk compared to phenotype 1 (sHR 0.67, 95% CI 0.47-0.94; *P*=.02). Although phenotypes 2 and 3 demonstrated trends toward lower risk, these associations were not statistically significant.

The risk of cardiovascular death was significantly lower in all phenotypes compared to phenotype 1: phenotype 2 (aHR 0.52, 95% CI 0.33-0.85; *P*=.008), phenotype 3 (aHR 0.57, 95% CI 0.32-0.99; *P*=.048), and phenotype 4 (aHR 0.49, 95% CI 0.33-0.72; *P*<.001). Similarly, all-cause death was significantly reduced in all phenotypes compared to phenotype 1: phenotype 2 (aHR 0.66, 95% CI 0.50-0.87; *P*=.003), phenotype 3 (aHR 0.44, 95% CI 0.30-0.65; *P*<.001), and phenotype 4 (aHR 0.56, 95% CI 0.44-0.71; *P*<.001).

Sensitivity analyses additionally adjusted for age; sex; hypertension; diabetes mellitus; heart failure; history of stroke, TIA, or thromboembolism; and vascular disease showed modest changes in several associations (Table S2 in Multimedia Appendix 1). For thromboembolic events, phenotype 2 became statistically significant (sHR 0.65, 95% CI 0.43-0.98; *P*=.04), while phenotype 3 was no longer significant. For MACE, phenotype 2 was significantly associated with lower risk (sHR 0.58, 95% CI 0.38-0.88; *P*=.01), whereas the association for phenotype 4 was attenuated but remained significant. Regarding cardiovascular death, phenotypes 2 and 3 lost statistical significance, while phenotype 4 remained significant (aHR 0.67, 95% CI 0.45-0.98; *P*=.04). For all-cause death, phenotype 2 was no longer significant, whereas phenotypes 3 and 4 remained associated with lower risk.

**Figure 6:**
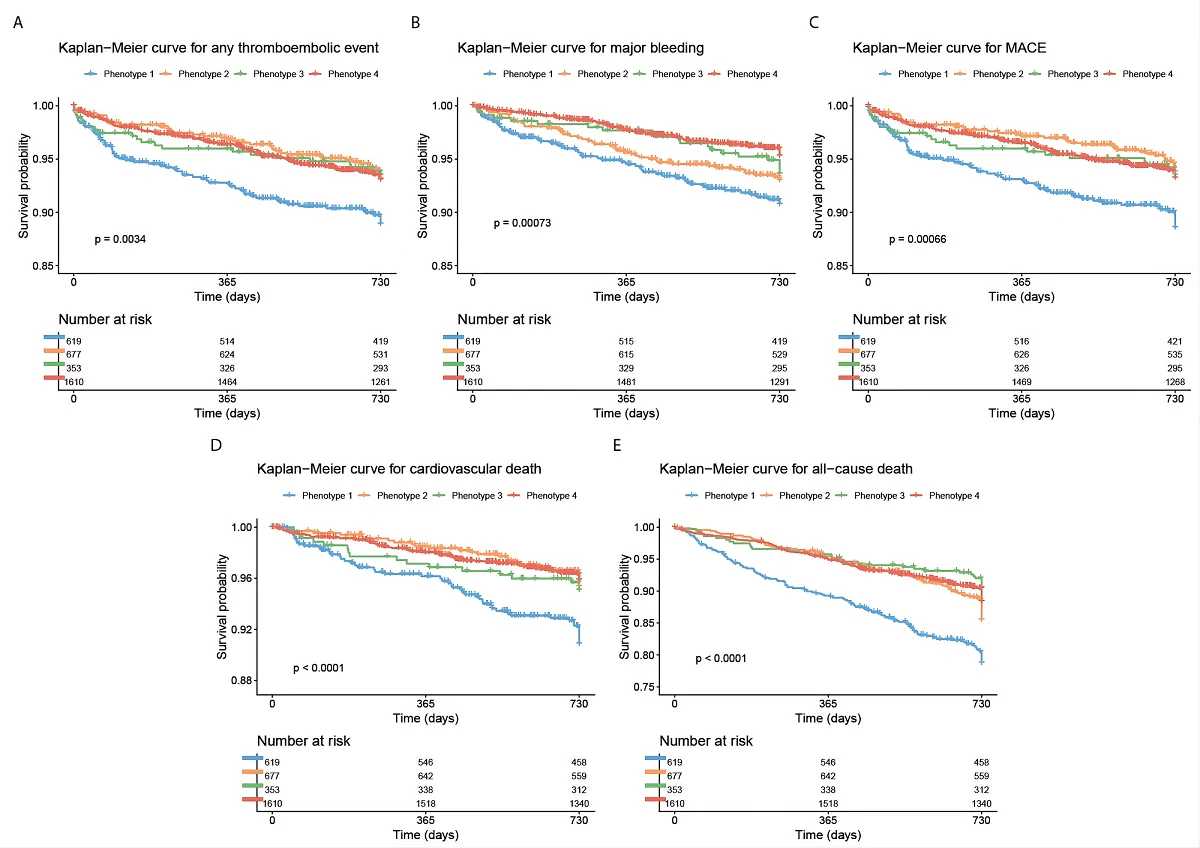
Kaplan-Meier survival curves for the primary clinical outcomes across the 4 generative topographic mapping (GTM)–derived AF phenotypes in the Murcia Atrial Fibrillation Project III (MAFP-III) cohort. Curves illustrate event-free survival for (A) thromboembolic events, (B) major bleeding, (C) major adverse cardiovascular events (MACE), (D) cardiovascular death, and (E) all-cause death. Color coding represents the final phenotype allocation: phenotype 1=blue; phenotype 2=orange; phenotype 3=green; and phenotype 4=red. Phenotype 1, older patients with highest cardiometabolic burden; phenotype 2, older patients with lower cardiometabolic risk; phenotype 3, comparatively younger patients with intermediate-high cardiometabolic risk; and phenotype 4, comparatively younger patients with intermediate cardiometabolic risk.

Kaplan-Meier survival analyses further supported these findings, showing significantly lower event-free survival in phenotype 1 for all outcomes, including thromboembolic events, major bleeding, MACE, cardiovascular death, and all-cause death, compared to the other phenotypes (log-rank *P*<.05 for all comparisons; Figure 6).

## Discussion

In this study, we applied a novel machine learning approach based on GTM to identify clinical phenotypes within the MAFP-III cohort. Our findings are twofold: (1) GTM proved highly effective in detecting clinically meaningful phenotypic subgroups, thereby enhancing patient stratification; and (2) these phenotypes were strongly associated with distinct prognostic outcomes. Its probabilistic framework enabled refined patient classification by managing uncertainty, capturing latent structures, and visualizing complex, high-dimensional data in a low-dimensional space, ultimately improving interpretability and supporting clinical decision-making.

This probabilistic framework also enables the identification of patients with lower maximum membership probabilities, typically located near cluster boundaries in the latent space. These individuals may represent transitional or overlapping phenotypes, reflecting more heterogeneous or evolving clinical profiles [11,12]. This may be particularly relevant in AF, where multimorbidity and dynamic risk trajectories are common. Such probabilistic information may therefore provide additional clinical value by capturing uncertainty and supporting more nuanced, personalized risk assessment, although this warrants further investigation.

From a clinical perspective, GTM complements existing risk scores such as CHA_2_DS_2_-VASc, CHA_2_DS_2_-VA, and HAS-BLED by integrating the broader cardiometabolic burden. This enables identification of patient subgroups simultaneously at high thromboembolic, bleeding, and cardiovascular risk profiles that may be overlooked when risks are considered separately. Such information could support more precise risk stratification, prioritize closer follow-up, optimize preventive strategies, and tailor therapeutic intensity in clinical practice [17,20].

In this context, GTM-derived phenotypes may provide complementary prognostic information beyond conventional risk scores by capturing complex, nonlinear interactions between cardiometabolic factors that are not fully reflected in additive scoring systems. This approach may be particularly valuable in refining risk stratification among patients with intermediate or overlapping risk profiles [10].

Beyond direct clinical application, GTM-based stratification may also enhance clinical research by enriching trial populations and identifying subgroups with differential treatment responses [21]. As electronic health records (EHRs) and decision-support tools expand, GTM could be embedded as an analytical layer, automatically classifying patients and translating complex data into actionable insights without increasing clinician workload.

From a digital health and implementation perspective, this framework could be operationalized within EHR systems to enable automated, real-time phenotypic assignment at the point of AF diagnosis. Once trained, the model allows projection of new patients into the latent space using routinely collected structured clinical data, facilitating scalable deployment and seamless integration into clinical workflows. Importantly, the probabilistic and visual nature of GTM may enhance interpretability and support clinician trust in AI-driven outputs. Future work should focus on prospective validation, interoperability with existing EHR infrastructures, and the development of user-centered clinical decision support tools [22].

Building upon this implementation framework, although phenotypes were derived from baseline characteristics, the GTM model also enables projection of longitudinal patient data onto the same latent space, allowing dynamic remapping as clinical profiles evolve over time [23]. This longitudinal extension may facilitate tracking of phenotypic trajectories and provide insights into disease progression and time-updated risk stratification. Future studies should incorporate repeated measurements and temporal validation to determine the clinical relevance of these dynamic phenotypic transitions.

Compared to traditional clustering methods such as hierarchical clustering and k-prototype, GTM showed superior performance in complex clinical datasets with mixed variable types [10,24]. Unlike approaches that impose rigid cluster boundaries or assume linear separability, GTM uncovers nonlinear relationships and accommodates the natural heterogeneity of AF populations [7,16,25,26]. Its probabilistic nature provides flexible clustering and a topologically coherent 2D representation, facilitating intuitive understanding of patient distributions and clinical trajectories [27].

In our cohort, GTM identified 4 distinct clinical phenotypes: (1) older patients with the highest cardiometabolic burden, (2) older patients with lower cardiometabolic risk, (3) comparatively younger patients with intermediate-high cardiometabolic risk, and (4) comparatively younger patients with intermediate cardiometabolic risk.

Phenotype 1 represented the least favorable reference profile, with the highest overall burden of adverse outcomes. After accounting for competing mortality risk, phenotype 4 demonstrated significantly lower risks of thromboembolic events, major bleeding, and MACE compared with phenotype 1, in addition to reduced cardiovascular and all-cause death. Phenotype 2 showed lower risks of thromboembolic events and MACE; however, its associations with cardiovascular and all-cause death were attenuated and no longer statistically significant after adjustment for age, sex, and comorbidities. Phenotype 3 was associated with lower all-cause death, although its associations with cardiovascular death and nonfatal outcomes were attenuated and less consistent after full adjustment. Age and cardiometabolic comorbidities, such as hypertension, diabetes, obesity, and vascular disease, play a central role in atrial remodeling, systemic inflammation, and endothelial dysfunction, thereby creating a prothrombotic milieu that increases the risk of thromboembolic events in AF [28,29]. This relationship was reflected in our cohort, where the phenotype characterized by the highest cardiometabolic burden (phenotype 1) consistently showed the least favorable prognosis, whereas the comparatively younger phenotype with intermediate cardiometabolic burden (phenotype 4) exhibited the most favorable overall outcome profile, particularly for thromboembolic events, major bleeding, MACE, and mortality. By integrating multidimensional clinical information, GTM-derived phenotypes capture the complex interplay between cardiometabolic status and prognosis, complementing conventional risk scores and refining risk stratification.

To the best of our knowledge, the only other study deriving AF phenotypes with a robust AI-based probabilistic approach was conducted by Bellfield et al [10]. Our findings build on and extend their work, showing that GTM can generate clinically meaningful phenotypes across diverse populations, with notable parallels to those reported in the UK Biobank and MIMIC-IV (Medical Information Mart for Intensive Care IV) cohorts. Although comparisons with the general AF cohort from the UK Biobank may be complex, we observed several similarities. For instance, our phenotype 1 appears analogous to their phenotype 3, characterized by older age and extensive cardiovascular and renal comorbidities. Similarly, our phenotype 2, comprising predominantly women with low cardiometabolic risk, mirrors their phenotype 5.

Moreover, comparisons with studies using traditional clustering techniques revealed partial concordance. For instance, Chen et al [7] applied hierarchical clustering to a cohort of 3348 patients with AF from the KERALA-AF Registry, identifying 5 distinct clinical phenotypes. Among these, Cluster 2, comprising patients aged older than 65 years with a high burden of multimorbidity, including cardiovascular-renal-metabolic syndrome, and Cluster 4, characterized by patients with heart failure and multiple comorbidities, appear comparable to our phenotypes 1 and 3, respectively. In their analysis, both clusters were associated with a significantly increased risk of MACE when compared with cluster 3, which included patients aged 65 years or younger with few comorbidities (cluster 2: odds ratio [OR] 1.79, 95% CI 1.42-2.25; cluster 4: OR 1.76, 95% CI 1.31-2.36).

Similarly, in the study by Bisson et al [30], conducted within the Loire Valley AF cohort, cluster 2—which included older patients with permanent AF, structural cardiac abnormalities, and a substantial comorbidity burden—resembles our phenotype 1. This cluster also showed a significantly elevated risk of all-cause death (hazard ratio 3.54, 95% CI 1.49-8.43) when compared with a reference cluster composed of patients with fewer comorbidities.

Altogether, our findings confirm that GTM is a powerful and reliable tool for uncovering actionable phenotypic patterns in AF, offering a pathway toward more personalized, prognosis-guided care. The identification of clinically meaningful and prognostically distinct phenotypes supports its potential integration into clinical workflows, where it could enable earlier identification of patients at high-risk and more precise allocation of preventive and therapeutic strategies. Future studies should validate these findings in independent cohorts and assess its use in real-time practice, as well as its added value when combined with longitudinal data and biomarkers. As health care moves toward precision medicine, GTM exemplifies how AI can translate complex data into actionable insights, bringing us closer to truly personalized AF management.

This study has several limitations. First, the nonrandomized nature of the study limits our ability to infer causality between phenotypes and outcomes. Although we adjusted for major confounders, residual confounding cannot be entirely excluded. Second, phenotypes were derived solely from baseline characteristics, without accounting for temporal changes. As risk factors evolve over time, some patients may shift phenotypic categories during follow-up. Although the GTM framework allows projection of new data onto the latent space, enabling potential dynamic remapping, this was not explored in the present study. Third, although we used rigorous internal validation to determine the optimal number of clusters, alternative methods or thresholds may yield different results. Furthermore, although internal cross-validation was used to optimize model performance, the identified phenotypes were not externally validated in an independent cohort. Therefore, the generalizability and reproducibility of these findings across independent AF populations remain to be established. Fourth, although key variables such as age and cardiometabolic comorbidities are central to AF pathophysiology and were intentionally included to enhance clinical relevance, we did not perform sensitivity analyses excluding these factors. Therefore, the robustness of the identified phenotypes under alternative modeling configurations remains uncertain and should be explored in future studies. Additionally, we did not include certain clinically relevant variables, such as cancer, in the phenotyping model. Although malignancy may have important prognostic implications [31], its heterogeneous nature and complex interactions with clinical management may limit its integration within a cardiometabolic-focused modeling framework. Future studies should explore the impact of incorporating such variables into GTM-based phenotyping. Fifth, all participants were already receiving OAC therapy. This may limit the applicability of findings to untreated AF populations or those on different therapeutic regimens. Sixth, the cohort was predominantly Caucasian, potentially restricting generalizability to more ethnically diverse populations. Given known disparities in AF outcomes by ethnicity, further validation in broader populations is warranted [32,33]. Finally, we did not perform direct head-to-head comparisons between GTM-derived phenotypes and established risk scores such as CHA_2_DS_2_-VASc and CHA_2_DS_2_-VA (for thromboembolism) or HAS-BLED (for bleeding). In addition, the applicability of GTM-based phenotyping in routine clinical practice remains exploratory and requires prospective validation before it can be integrated into decision-making.

In conclusion, this clustering analysis using GTM identified 4 distinct clinical phenotypes among patients with AF receiving oral anticoagulation, each associated with different prognostic profiles. GTM enabled the modeling of complex, nonlinear relationships within high-dimensional clinical data, facilitating stratification into clinically meaningful phenotypic subgroups. These findings support the potential utility of GTM-based phenotyping for improving risk characterization in AF. However, external validation studies are required to determine the generalizability and clinical applicability of these phenotypes.

## Data Availability

All data generated or analyzed during this study are included in this published paper. Additional derived data supporting the findings are available from the corresponding author upon reasonable request.

## Appendix

Multimedia Appendix 1 Additional tables.

## Abbreviations

AF: atrial fibrillation
aHR: adjusted hazard ratio
CHA2DS2-VA: Congestive heart failure, Hypertension, Age ≥ 75 [doubled score], Diabetes, Stroke [doubled], Vascular disease, Sex category [female]
CHA2DS2-VASc: Congestive heart failure, Hypertension, Age ≥ 75 [doubled score], Diabetes, Stroke [doubled], Vascular disease
COPD: chronic obstructive pulmonary disease
DOAC: direct-acting OAC
EHR: electronic health record
GTM: generative topographic mapping
HAS-BLED: Hypertension, Abnormal renal and liver function, Stroke, Bleeding, Labile INR, Elderly, Drugs or alcohol
ISTH: International Society on Thrombosis and Haemostasis
MACE: major adverse cardiovascular events
MAFP-III: Murcia Atrial Fibrillation Project III
MIMIC-IV: Medical Information Mart for Intensive Care IV
NLL: negative log-likelihood
OAC: oral anticoagulation
OR: odds ratio
OSA: obstructive sleep apnea
sHR: subdistribution hazard ratio
TIA: transient ischemic attack
VKA: vitamin K antagonist
