# Supplementary material for: AI-Based Phenotyping of Atrial Fibrillation Through Generative Topographic Mapping: Prospective Murcia Atrial Fibrillation Project III Cohort Study
Source: J Med Internet Res. 2026 Aug 6;28:e90502. doi: 10.2196/90502 (PMC13494521; doi:10.2196/90502)
Supplement: Supplementary file 1 [file jmir_v28i1e90502_app1.docx]

| **Supplementary Material**  **Table S1.** Post hoc pairwise comparison *P*-values for baseline clinical characteristics across the four GTM-derived AF phenotypes identified in the MAFP-III cohort, a prospective observational study of anticoagulated patients with newly diagnosed AF recruited in Murcia, Spain, between January 2016 and November 2021. | | | | |  |
| --- | --- | --- | --- | --- | --- |
|  | **Phenotype 1**  **(N = 619)** | **Phenotype 2**  **(N = 677)** | **Phenotype 3**  **(N = 353)** | **Phenotype 4**  **(N = 1,610)** |  |
| **MODELING VARIABLES** | | | | |  |
| **Demographics, median [IQR]** | | | | |  |
| Weight (kg) | [Ref.] | <.001 | <.001 | .37 |  |
| Abdominal circumference (cm) | [Ref.] | <.001 | <.001 | <.001 |  |
| Body fat (%) | [Ref.] | <.001 | <.001 | <.001 |  |
| **Blood count parameters, median [IQR]** | | | | |  |
| Hemoglobin (g/dL) | [Ref.] | <.001 | <.001 | <.001 |  |
| Leukocyte count (x10^9^/L) | [Ref.] | <.001 | <.001 | <.001 |  |
| Platelet count (x10^9^/L) | [Ref.] | <.001 | <.001 | <.001 |  |
| **Diabetes risk markers, median [IQR]** | | | | |  |
| Glucose (mg/dL) | [Ref.] | <.001 | <.001 | <.001 |  |
| HbA1c | [Ref.] | <.001 | <.001 | <.001 |  |
| **Renal function, median [IQR]** | | | | |  |
| Creatinine (mg/dL) | [Ref.] | <.001 | <.001 | <.001 |  |
| **Liver function, median [IQR]** | | | | |  |
| Aspartate aminotransferase | [Ref.] | .001 | <.001 | <.001 |  |
| Alanine aminotransferase | [Ref.] | <.001 | <.001 | <.001 |  |
| **Cholesterol markers, median [IQR]** | | | | |  |
| Cholesterol (mg/dL) | [Ref.] | .32 | <.001 | <.001 |  |
| HDL cholesterol (mg/dL) | [Ref.] | <.001 | <.001 | <.001 |  |
| Triglycerides (mg/dL) | [Ref.] | <.001 | <.001 | .46 |  |
| **Arterial blood pressure, median [IQR]** | | | | |  |
| Systolic BP (mmHg) | [Ref.] | .004 | <.001 | <.001 |  |
| Diastolic BP (mmHg) | [Ref.] | <.001 | <.001 | <.001 |  |
| **Echocardiographic markers, median [IQR]** | | | | |  |
| LVEF (%) | [Ref.] | .06 | .18 | .38 |  |
|  | | | | |  |
| **ADDITIONAL INVESTIGATIVE VARIABLES** | | | | |  |
|  | | | | |  |
| **Demographics** | | | | |  |
| Age (years), median [IQR] | [Ref.] | .33 | <.001 | <.001 |  |
| Sex [Female], n (%) | [Ref.] | .91 | .02 | .002 |  |
| BMI (kg/m^2^), median [IQR] | [Ref.] | <.001 | <.001 | <.001 |  |
| **Type of AF, n (%)** | [Ref.] | .82 | .09 | .09 |  |
| **Comorbidities, n (%)** | | | | |  |
| Hypertension | [Ref.] | <.001 | .07 | .002 |  |
| Diabetes mellitus | [Ref.] | <.001 | <.001 | <.001 |  |
| Heart failure | [Ref.] | <.001 | 0.99 | .001 |  |
| History of stroke/TIA/thromboembolism | [Ref.] | .85 | .21 | .018 |  |
| Vascular disease* | [Ref.] | .001 | <.001 | <.001 |  |
| Renal impairment | [Ref.] | <.001 | <.001 | <.001 |  |
| Hypercholesterolemia | [Ref.] | <.001 | .009 | .002 |  |
| COPD/OSA | [Ref.] | <.001 | <.001 | .001 |  |
| History of relevant bleeding | [Ref.] | .27 | .18 | <.001 |  |
| Liver disease | [Ref.] | .69 | .15 | .73 |  |
| History of cancer | [Ref.] | .36 | .18 | .27 |  |
| Smoking habit | [Ref.] | .08 | .51 | .53 |  |
| Alcoholism | [Ref.] | .12 | .60 | .04 |  |
|  | | | | |  |
| **CONCOMITANT TREATMENT, n (%)** | | | | |  |
|  | | | | |  |
| DOAC | [Ref.] | <.001 | .001 | .10 |  |
| VKA | [Ref.] | <.001 | .001 | .10 |  |
| Antiarrhythmics | [Ref.] | .44 | .78 | .74 |  |
| ACE inhibitors | [Ref.] | .57 | .82 | .09 |  |
| ARBs | [Ref.] | .22 | .003 | .92 |  |
| Calcium channel blockers | [Ref.] | .003 | .10 | .002 |  |
| Beta-blockers | [Ref.] | .53 | .26 | .21 |  |
| Diuretics | [Ref.] | <.001 | .15 | <.001 |  |
| Antilipemic agents | [Ref.] | <.001 | .03 | .001 |  |
| Oral hypoglycemic agents | [Ref.] | <.001 | <.001 | <.001 |  |
| Insulins | [Ref.] | <.001 | <.001 | <.001 |  |
| Antiplatelet therapy | [Ref.] | <.001 | .002 | .001 |  |
|  | | | | |  |
| **STROKE AND BLEEDING SCORES, median [IQR]** | | | | |  |
| CHA_2_DS_2_-VASc | [Ref.] | <.001 | <.001 | <.001 |  |
| CHA_2_DS_2_-VA | [Ref.] | <.001 | <.001 | <.001 |  |
| HAS-BLED | [Ref.] | <.001 | <.001 | <.001 |  |
| AF = atrial fibrillation; ACE inhibitors = angiotensin-converting enzyme inhibitors; ARBs = angiotensin II receptor blockers; BMI = body mass index; BP = blood pressure; COPD/OSA = chronic obstructive pulmonary disease/obstructive sleep apnea; DOAC = direct oral anticoagulants; HbA1c = glycosylated hemoglobin; HDL = high-density lipoproteins; IQR = interquartile range; LVEF = left ventricular ejection fraction; Ref = reference; TIA = transient ischemic attack; VKA = vitamin K antagonists.  *Vascular disease includes coronary artery disease and/or peripheral artery disease. | | | | |  |

|  | **Table S2.** Association between GTM-derived AF phenotypes and clinical outcomes in the MAFP-III cohort. Adjusted Fine–Gray competing risk models were used for non-fatal outcomes (thromboembolic events, major bleeding, and MACE), with death treated as a competing event, whereas adjusted Cox proportional hazards models were used for cardiovascular death and all-cause death. | | | | | | |
| --- | --- | --- | --- | --- | --- | --- | --- |
|  |  | **Adjusted model**** | | | | | |
|  |  | **Hazard Ratio*** | | **95% CI** | ***P*** | | |
|  | **Any thromboembolic event** |  | |  |  | | |
| Phenotype 1 (N = 619) | | **[Ref.]** | |  |  | | |
|  | Phenotype 2 (N = 677) | 0.65 | 0.43-0.98 | | | .04 |  |
|  | Phenotype 3 (N = 353) | 0.70 | 0.42-1.17 | | | .17 |  |
|  | Phenotype 4 (N = 1,610) | 0.70 | 0.50-0.99 | | | .049 |  |
|  | **Major bleeding** |  | |  |  | | |
|  | Phenotype 1 (N = 619) | **[Ref.]** | |  |  | | |
|  | Phenotype 2 (N = 677) | 0.86 | | 0.57-1.32 | .49 | | |
|  | Phenotype 3 (N = 353) | 0.85 | | 0.51-1.44 | .56 | | |
|  | Phenotype 4 (N = 1,610) | 0.60 | | 0.41-0.88 | .01 | | |
|  | **MACE** |  | |  |  | | |
|  | Phenotype 1 (N = 619) | **[Ref.]** | |  |  | | |
|  | Phenotype 2 (N = 677) | 0.58 | | 0.38-0.88 | .01 | | |
|  | Phenotype 3 (N = 353) | 0.70 | | 0.42-1.17 | .18 | | |
|  | Phenotype 4 (N = 1,610) | 0.69 | | 0.50-0.97 | .03 | | |
|  | **Cardiovascular death** |  | |  |  | | |
|  | Phenotype 1 (N = 619) | **[Ref.]** | |  |  | | |
|  | Phenotype 2 (N = 677) | 0.67 | | 0.41-1.08 | .10 | | |
|  | Phenotype 3 (N = 353) | 0.77 | | 0.44-1.45 | .36 | | |
|  | Phenotype 4 (N = 1,610) | 0.67 | | 0.45-0.98 | .04 | | |
|  | **All-cause death** |  | |  |  | | |
|  | Phenotype 1 (N = 619) | **[Ref.]** | |  |  | | |
|  | Phenotype 2 (N = 677) | 0.76 | | 0.58-1.01 | .10 | | |
|  | Phenotype 3 (N = 353) | 0.61 | | 0.41-0.90 | .01 | | |
|  | Phenotype 4 (N = 1,610) | 0.73 | | 0.58-0.93 | .01 | | |
|  | 95%CI = 95% confidence interval; aHR= adjusted hazard ratio; sHR = subdistribution hazard ratio; MACE = major adverse cardiovascular events; Ref = reference.  * sHR are reported for non-fatal outcomes, while aHR are reported for cardiovascular and all-cause death.  **Adjusted model by age, sex, hypertension, diabetes mellitus, heart failure, stroke/TIA/thromboembolism and vascular disease. | | | | | | |
